# Wireless Body Area Networks: UWB Wearable Textile Antenna for Telemedicine and Mobile Health Systems

**DOI:** 10.3390/mi11060558

**Published:** 2020-05-30

**Authors:** Ashok Yadav, Vinod Kumar Singh, Akash Kumar Bhoi, Gonçalo Marques, Begonya Garcia-Zapirain, Isabel de la Torre Díez

**Affiliations:** 1 Department of ECE, Krishna Engineering College, Ghaziabad 201007, India; ashok.biet@gmail.com; 2 Department of Electrical Engineering, S.R. Group of Institutions, Jhansi 284002, U.P., India; singhvinod34@gmail.com; 3 Department of Electrical and Electronics Engineering, Sikkim Manipal Institute of Technology, Sikkim Manipal University, Majhitar 737136, Sikkim, India; 4 Instituto de Telecomunicações, Universidade da Beira Interior, 6201-001 Covilhã, Portugal; goncalosantosmarques@gmail.com; 5 eVIDA Research Group, University of Deusto. Avda/Universidades 24, 48007 Bilbao, Spain; 6 Department of Signal Theory and Communications, and Telematics Engineering University of Valladolid, Paseo de Belén 15, 47011 Valladolid, Spain; isator@tel.uva.es

**Keywords:** textile antenna, ultra-wideband, circuit theory, frequency domain, time domain, SAR

## Abstract

A compact textile ultra-wideband (UWB) antenna with an electrical dimension of 0.24λ_o_ × 0.24λ_o_ × 0.009λ_o_ with microstrip line feed at lower edge and a frequency of operation of 2.96 GHz is proposed for UWB application. The analytical investigation using circuit theory concepts and the cavity model of the antenna is presented to validate the design. The main contribution of this paper is to propose a wearable antenna with wide impedance bandwidth of 118.68 % (2.96–11.6 GHz) applicable for UWB range of 3.1 to 10.6 GHz. The results present a maximum gain of 5.47 dBi at 7.3 GHz frequency. Moreover, this antenna exhibits Omni and quasi-Omni radiation patterns at various frequencies (4 GHz, 7 GHz and 10 GHz) for short-distance communication. The cutting notch and slot on the patch, and its effect on the antenna impedance to increase performance through current distribution is also presented. The time-domain characteristic of the proposed antenna is also discussed for the analysis of the pulse distortion phenomena. A constant group delay less than 1 ns is obtained over the entire operating impedance bandwidth (2.96–11.6 GHz) of the textile antenna in both situations, i.e., side by side and front to front. Linear phase consideration is also presented for both situations, as well as configurations of reception and transmission. An assessment of the effects of bending and humidity has been demonstrated by placing the antenna on the human body. The specific absorption rate (SAR) value was tested to show the radiation effect on the human body, and it was found that its impact on the human body SAR value is 1.68 W/kg, which indicates the safer limit to avoid radiation effects. Therefore, the proposed method is promising for telemedicine and mobile health systems.

## 1. Introduction

The communications and telemedicine industry have been continuously evolving over the years. Wearable compact antennas constitute a significant part of every wearable communication for telemedicine systems. The wideband characteristic of wearable antennas is critical for telemedicine and mobile healthcare systems. In healthcare scenarios, the integration of antennas with a human body is essential to promote continuous monitoring. However, these processes arise due to deformation on different angles in the human body and biological material properties. In such a situation, wearable antennas have crucial advantages. The textile-based Ultra-wideband (UWB) antennas have vast applications in telemedicine and healthcare scenarios. It can be used for short-distance communication with high-speed data and low power consumption for continuous real-time monitoring of psychological patients’ data. In healthcare monitoring systems, Wireless Body Area Networks (WBAN) require wide bandwidth to guarantee continuous patient supervision, therefore, wearable and compact antennas play a crucial role in the design and development of telemedicine systems.

UWB came into existence in the year 2002 and allowed the frequency spectrum of 3.1–10.6 GHz [1]. This frequency spectrum was given by the Federal Communications Commission (FCC). The UWB antenna is very popular owing to a simple and compact design, low profile and high-speed data transfer rate for short-range communication with low power consumption. The telemedicine and mobile health domains have encouraged multiple studies on the design and development of UWB antennas [2,3,4,5,6,7,8,9]. Therefore, numerous design configurations of UWB antennas have been presented such as rectangular, circular and slot configurations [10,11,12,13]. These antennas send signal information in the form of short pulses at a specific time interval to reduce the effect of multipath fading. 

Nowadays, wearable devices are very popular in the monitoring of human body activity such as sports, battlefields, workouts and healthcare systems [14,15,16]. The UWB technology is being used extensively due to its essential features, such as low cost, low-power consumption and high data rate [17]. Apart from this, power spectral density over the entire UWB band is very low, which provides less interference [18]. Besides this, the effect of radiation on the human body is also satisfactory. Therefore, this technology has been considered very promising for wearable applications all over the world.

Furthermore, when wearable and flexible antennas are compared with conventional rigid antennas, they are characterized in terms of compactness, flexibility, durability and reconfiguration. The fabrication process and material selection play a critical role to implement that kind of antennas. Moreover, additional measurement setup and tuning process are needed to reflect the practical design and optimal performance of wearable and flexible antennas [19]. Textile materials are used as conductive materials or substrate materials that are required for application of clothing integration. It has a low relative permittivity (ε_r_), low loss tangent (tan (δ)) and slimmer thickness (h), which reduce the surface wave losses and enhance the performance of antenna parameters [20]. 

From the design point of view, it is possible to characterize the wearable textile antenna design procedures in four different considerations. In the first, the material is selected according to the design requirement by including conducting and substrate material. For the second step, the uncharacterized material must be studied according to its electrical properties, such as dielectric constant, loss tangent and conductivity of the material. Based on the given information, radiation element and feed methods of wearable textile antennas are simulated, optimized and designed in the third step before fabrication. Finally, in the fourth step, the selection of the fabrication method is conducted as per antenna structure and material.

In addition to conventional measurement testing, such as return loss, radiation pattern, gain, and qualitative tests should be conducted to fulfill the necessity of wearable applications [21]. These measurements include the specific absorption rate (SAR) analysis, humidity, bending effect, thermal and robustness test, which is not often used in other conventional antennas applications. The UWB antennas, designed with fabric like jeans and cotton or perforated plastic as a substrate, can be used as a wearable device on the human body due to its low effect on the human body [22,23,24,25,26].

The main contribution of this work is to present a novel compact UWB antenna on a fabric jeans (ε_r_ = 1.7) substrate, with microstrip line feed and the partial ground. The wearable textile UWB antenna includes a radiation patch with the modified ground and executes excellent radiation characteristic in the frequency range of 3.1 to 10.6 GHz. Real-time on-body measurement is used to show the antenna performance due to the bending and humidity effect.

The entire paper is organized in five different sections with an introduction to cover in Section 1. The UWB antenna configuration is presented in Section 2, whereas parametric analysis is in Section 3. The results and discussion are presented in Section 4. Finally, the conclusion is given in Section 5.

## 2. Materials and Methods 

This section presents the textile antenna design and theoretical analysis. The textile material has low dielectric constant, which reduces the surface wave losses occur in the antenna. The wearable antennas’ application should be incorporated with the human body. However, due to the human body’s shape and electrical properties of biological tissues, the antenna’s functioning is affected. The rigid materials with high dielectric constant fail these criteria, and low-dielectric-constant materials came into the picture to fulfil all these requirements. The low-dielectric materials also play a critical role for bandwidth enhancement and antennas flexibility. Therefore, the optimization process is proposed to justify the UWB antenna design. Furthermore, the mathematical circuit theory analysis is also performed to verify the function of the UWB textile antenna. As a result, the following subsections describe the material and designing methods. 

### 2.1. Textile Antenna Design

**Figure 1:**
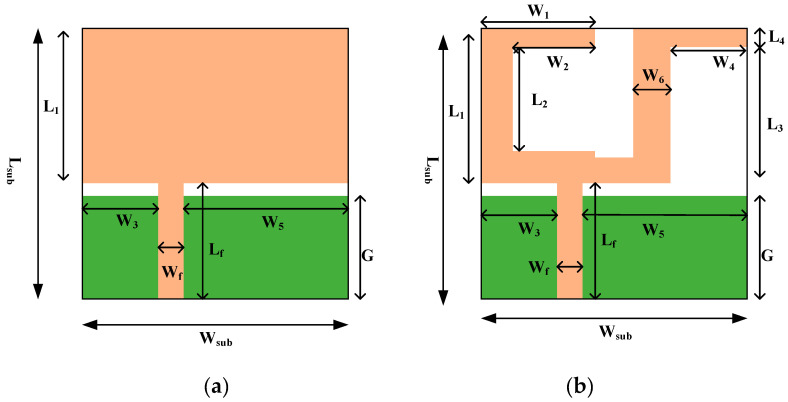
(**a**) Layout of simple RSMA (**b**) and optimized antenna design.

Figure 1a,b shows the rectangular patch and design of the proposed textile antenna in the UWB range. The antenna is made of a copper patch sandwiched with jeans fabric, where the upper patch is the radiating element and the lower patch is the ground plane and the jeans’ fabric is the substrate. The jeans’ fabric has low dielectric constant (ε_r_ = 1.7), and that reduces the surface wave losses by improving the impedance of the textile antenna. Therefore, it is used as a substrate in the antenna design. Furthermore, a 3.5 × 10 × 1 mm^3^ microstrip line is used to provide excellent impedance matching of 50 Ω in the designed antenna.

The proposed antenna design came out from the optimization process in HFSS (High Frequency Structure Simulator) simulator. Table 1 describes the optimized geometrical parameters of the textile antenna.

**Figure 2:**
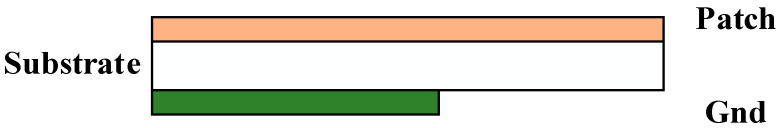
Side view of the antenna.

Moreover, Figure 2 visualizes the side view layout of textile antenna used in this manuscript.

The basic structure of the textile antenna includes a radiating patch with asymmetric microstrip line feed and a partial ground plane. The final structure is obtained through the evolution process, in which various activities of optimization process (patch length-width variation, slot, notch and ground variation) have been performed by HFSS simulator under parametric study. The use of slotting has been verified by reducing the shape and size of the radiating element and ground plane, while the full ground plane of the antenna has an effect on its impedance characteristic. The impedance matching has been improved by cutting a slot (W_2_ × L_2_), two notches (W_4_ × L_3_) and W_sub_ − (W_1_ + W_6_ + W_4_) × (L_4_ + L_2_ + 1). The best impedance matching is achieved by adjusting feed position (W_f_) and ground plane (G) and, therefore, the antenna’s performance is improved. 

**Figure 3:**
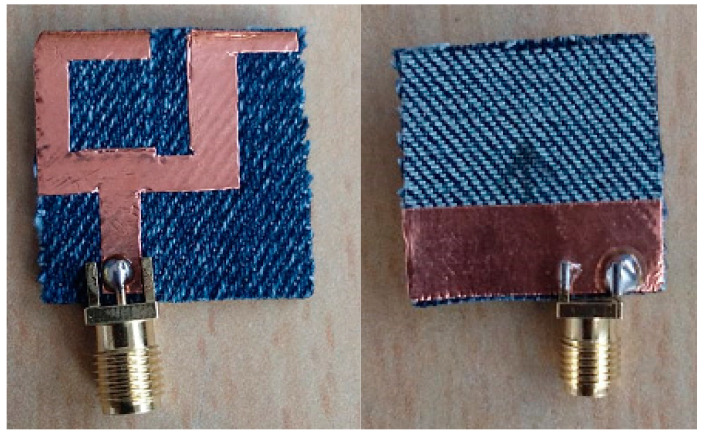
Optimized textile ultra-wideband (UWB) fabricated antenna.

On the one hand, by applying the iteration process to the primary antenna, the final design was obtained in which good impedance matching was achieved compared to the primary antenna. On the other hand, it can be observed that with a variation in a different slot, notch, patch and justified partial ground plane, the antenna reveals broad impedance bandwidth from 2.96 to 11.6 GHz (118.68%) with maximum resonance at 7.3 GHz frequency. As a result, the proposed antenna contains the complete 3.1–10.6 GHz range (UWB range) designated by FCC. Furthermore, the finalized textile antenna has been fabricated on jeans textile substrate including copper as a perfect electric conductor with loss tangent (tan (δ) = 0.025), thickness of 0.035 mm, ε_r_ = 1.7 and h = 1 mm. A 50 Ω SMA connector is joined via microstrip line feed to energize the antenna as illustrated in Figure 3.

### 2.2. Theoretical Analysis of Textile Antenna

The electrical equivalent circuit of a simple rectangular patch antenna is the parallel combination of resistance (R_1_), inductance (L_1_) and capacitance (C_1_) and their parameter values are given by [27].

(1) $$C_{1}=\frac{\varepsilon_{0}\varepsilon_{e}\mathrm{LW}}{2h}\cos^{-2}\left(\frac{{\pi y}_{0}}{L}\right)\;$$

(2) L1=1ω2C1

(3) R1=QrωC1

(4) εe=εr+12+εr−12(1+10hW)−12

where $\varepsilon_{r}$ = relative permittivity and $\varepsilon_{e}$ = effective permittivity, h = height of substrate.

(5) $$Q_{r}=\frac{c\sqrt{\varepsilon_{e}}}{4\mathrm{fh}}$$

where $Q_{r}$ = quality factor, f = resonating frequency and c = speed of light.

The impedance of a simple patch can be calculated as the following manner, which is presenting in Equation (6).

(6) $$Z_{\mathrm{Patch}}=\frac{1}{\frac{1}{R_{1}}+\frac{1}{{j\omega L}_{1}}+{j\omega C}_{1}}$$

This antenna is excited by a 50-ohm microstrip line feed, and its parameter values are evaluated by Equations (7) and (8) [28].

(7) $$C_{a}=W_{1}\left[\left(10.1{\log\varepsilon }_{r}+2.33\right)x\frac{W_{1}}{W_{2}}-12.6{\log\varepsilon }_{r}-317\right]\mathrm{pF}$$

(8) $$L_{a}=H\left[40.5\left(\frac{W_{1}}{W_{2}}-1\right)-75\log\left(\frac{W_{1}}{W_{2}}\right)+0.2{\left(\frac{W_{1}}{W_{2}}-1\right)}^{2}\right]\mathrm{nH}$$

In the optimized design, a slot was removed from the patch, and it makes a series combination of resistance of slot and reactance of slot, which are given in Equations (9)–(11) [29].

(9) $$Z_{\mathrm{slot}}=R_{\mathrm{slot}}+{\mathrm{jX}}_{\mathrm{slot}}$$

(10) $$R_{\mathrm{slot}}=60C+\ln\left({\mathrm{kL}}_{\mathrm{sl}}\right)+\frac{1}{2}\sin\left({\mathrm{kL}}_{\mathrm{sl}}\right)\left[S_{i}\left(2{\mathrm{kL}}_{\mathrm{sl}}\right)2S_{i}\left({\mathrm{kL}}_{\mathrm{sl}}\right)\right]+\frac{1}{2}\cos\left({\mathrm{kL}}_{\mathrm{sl}}\right)C+\ln\frac{{\mathrm{kL}}_{\mathrm{sl}}}{2}\;+C_{i}\left(2{\mathrm{kL}}_{\mathrm{sl}}\right)2C_{i}\left({\mathrm{kL}}_{\mathrm{sl}}\right)$$

(11) $$X_{\mathrm{slot}}=30\cos^{2}\alpha \left\{2S_{i}\left({\mathrm{kL}}_{\mathrm{sl}}\right)\;+\cos\left({\mathrm{kL}}_{\mathrm{sl}}\right)\left[2S_{i}\left({\mathrm{kL}}_{\mathrm{sl}}\right)-S_{i}\left(2{\mathrm{kL}}_{\mathrm{sl}}\right)-\sin\left({\mathrm{kL}}_{\mathrm{sl}}\right)\right]\left[2C_{i}\left({\mathrm{kL}}_{\mathrm{sl}}\right)-C_{i}\left(2{\mathrm{kL}}_{\mathrm{sl}}\right)\;-C_{i}\left(\frac{2{{\mathrm{kW}}^{2}}_{\mathrm{sl}}}{L_{\mathrm{sl}}}\right)\right]\right\}$$

where

$$S_{i}\left(x\right)=\int_{0}^{x}\frac{\sin\left(x\right)}{x}\mathrm{dx}\;\mathrm{and}\;C_{i}\left(x\right)=-\int_{x}^{\infty }\frac{\sin\left(x\right)}{x}\mathrm{dx}$$

$L_{\mathrm{sl}}=\mathrm{length}\;\mathrm{of}\;\mathrm{slot}$, C = 0.5772 (Euler’s constant) and k = propagation constant.

Cutting a slit on the edges of a patch is considered as a notch, which includes a parallel combination of R_2_, L_2_ and C_2_ whereas L_2_ is the series inductance value of $L_{1},$ ${\Delta L}_{1}$ and ${\Delta L}_{2}$. Similarly, C_2_ is also the series capacitance value of $C_{1},\;\Delta C_{1}\;\mathrm{and}\;\Delta C_{2}$ [30]. The additional inductance and capacitance values are evaluated by Equations (12)–(15). The total impedance due to two notches is calculated by using Equation (16).

(12) $$L_{2}=L_{1}+{\Delta L}_{1}+{\Delta L}_{2}$$

(13) $$C_{2}=\frac{C_{1}{\Delta C}_{1}{\Delta C}_{2}}{C_{1}+{\Delta C}_{1}+{\Delta C}_{2}}$$

(14) $$\Delta L=\frac{{h\mu }_{0}\pi }{8}\left(\frac{W_{\mathrm{patch}}}{w_{\mathrm{notch}}}\right)$$

(15) $$\Delta C=\left(\frac{W_{\mathrm{patch}}}{w_{\mathrm{notch}}}\right)C_{\mathrm{gap}\;\mathrm{capacitance}}$$

(16) $$Z_{\mathrm{notch}}=\frac{1}{\frac{1}{R_{2}}+\frac{1}{{j\omega L}_{2}}+{j\omega C}_{2}}$$

When a patch is included with a notch, coupling capacitance is introduced in the equivalent circuit of the proposed antenna, which is given in Equations (17) and (18), and gives its impedance value [31].

(17) $$C_{c}=\frac{-\left(C_{1}+C_{2}\right)+\sqrt{{\left(C_{1}+C_{2}\right)}^{2}-C_{1}C_{2}\left(1-\frac{1}{{k^{2}}_{c}}\right)}}{2}$$

where $k_{c}$ = coupling coefficient

(18) $$Z_{c}=\frac{1}{{j\omega C}_{c}}$$

The total impedance between notch and slot is evaluated by parallel combination in it and when coupling impedance is considered. Therefore, a combination series of Z and Z_C_ is obtained for total impedance. Finally, the input impedance is calculated by the parallel combination of $Z_{\mathrm{patch}}$ and $\left(Z_{c}+Z\right)$. The reflection coefficient, voltage standing wave ratio (VSWR) and return loss values are evaluated by using Equations (21)–(23), respectively.

(19) $$Z=\frac{Z_{\mathrm{notch}}Z_{\mathrm{slot}}}{Z_{\mathrm{notch}}+Z_{\mathrm{slot}}}$$

(20) $$Z_{\mathrm{in}}=\frac{Z_{\mathrm{patch}}\left(Z_{c}+Z\right)}{Z_{\mathrm{patch}}+Z_{c}+Z}$$

(21) $$Z_{\mathrm{in}}=\frac{Z_{\mathrm{patch}}\left(Z_{c}+Z\right)}{Z_{\mathrm{patch}}+Z_{c}+Z}$$

(22) $$\Gamma =\frac{Z_{0}-Z_{\mathrm{in}}}{Z_{0}+Z_{\mathrm{in}}}$$

(23) $$\mathrm{VSWR}=\frac{1+\left|\Gamma \right|}{1-\left|\Gamma \right|}$$

(24) $${\mathrm{Return}\;\mathrm{Loss}}_{\mathrm{dB}}=20\log\left|\Gamma \right|$$

**Figure 4:**
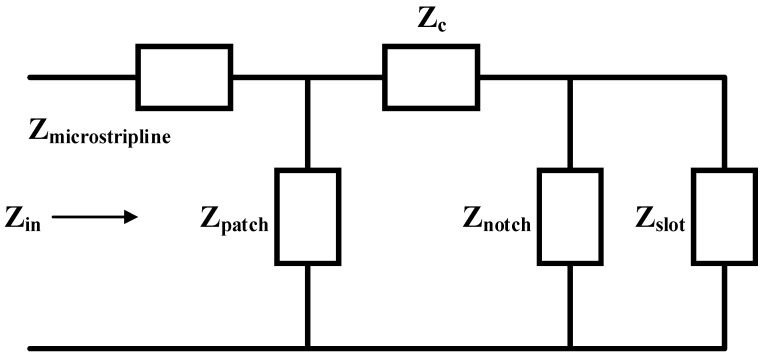
Equivalent circuit model of the proposed textile antenna.

Figure 4 represents the equivalent circuit of the proposed antenna. Furthermore, this design is also verified by circuit theory analysis through advanced design system (ADS), a key sight circuit simulator, and electrical parameters are evaluated.

## 3. Parametric Study of Textile UWB Antenna

The parametric analysis has been done to provide complete information about the design of the antenna. The variation in the radiating patch, cut slot, substrate, feed width and ground of any antenna causes variations in its dimensions as well as electrical parameters. Therefore, the performance parameters of the antenna are also changed. Different parametric studies have been used to analyze the antenna performance. 

The primary antenna was passed through different iterations in patch and ground to find out the proposed design, which produces various wideband performances. Multiple slots and notches have removed from the radiating patch and presented a justified ground plane by a partial ground modification to provide a reliable impedance matching. The feed width of 3.5 mm provides the best impedance matching with stable radiation properties.

**Figure 5:**
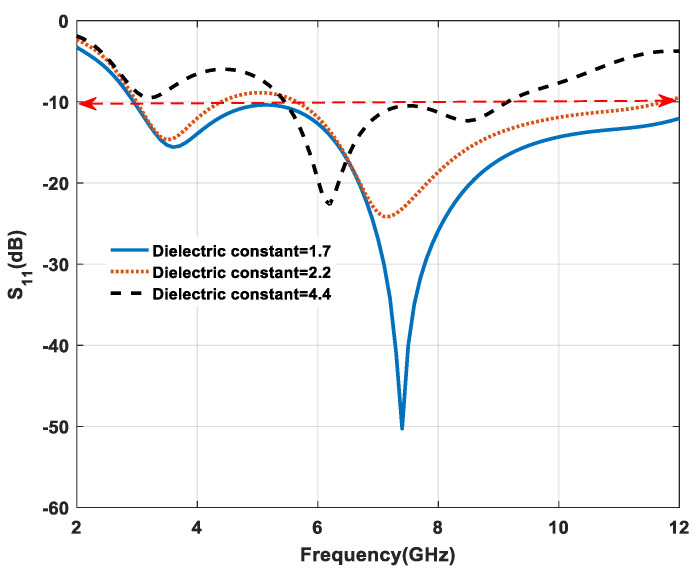
Effect of variation on dielectric constant.

The impedance matching is also affected by the variation in substrate material property, and it changes to the shifting of the characteristic impedance of the microstrip feed from 50 Ω. The proposed antenna performance is analyzed with three substrates with the same dimensions, as shown in Figure 5. In the case of 2.2 and 4.4 return loss (S_11_) and bandwidth are substantially affected. However, jeans substrate offers more bandwidth (2.96–12 GHz) among all three substrates because low-dielectric-constant material reduces the surface wave loss to improve impedance bandwidth. The best impedance matching is obtained at dielectric constant of 1.7.

**Figure 6:**
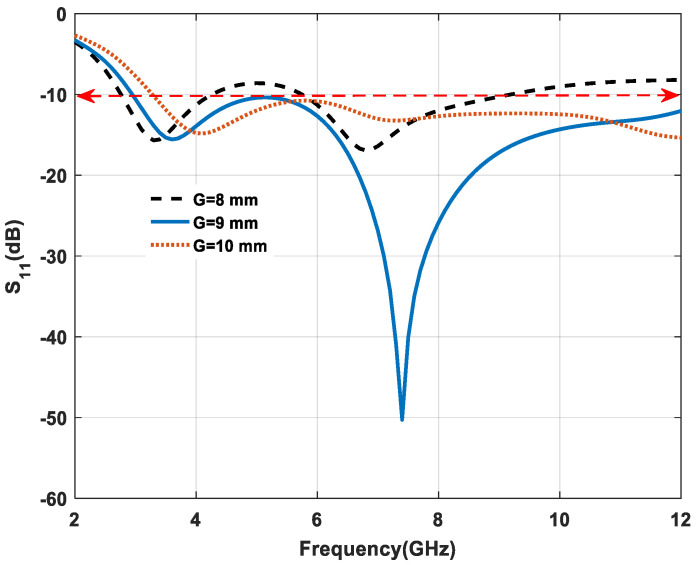
Effect of variation on a ground plane.

A significant variation in the ground plane is effected on antenna impedance matching at higher frequencies than at lower frequencies. By changing the ground plane from 8 mm to 10 mm with a step of 1 mm, proper matching is achieved at a partial ground plane value of 9 mm, which is shown in Figure 6. For ground plane (G) values of 8 and 10 mm, the resonance frequency is shifted from higher to lower value and return loss of the antenna is increased. Moreover, the bandwidth of the antenna is reduced. In both cases (8 and 9 mm), the return loss of the antenna is increased, which, in turn, can degrade the antenna performance. Therefore, a ground size of 9 mm is selected as the optimum size for a bandwidth of 120.85% (2.96–12 GHz).

**Figure 7:**
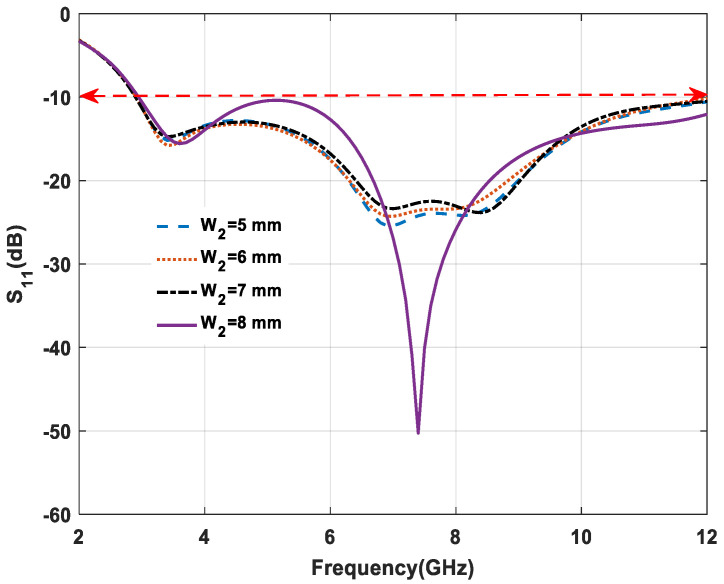
Effect of variation on W_2._

The coupling capacitor plays a critical role in controlling the resonance frequency and return loss (S_11_) as shown in Figure 7. With the increase of notch width, the effect of the coupling capacitor (C_C_) is analyzed. From 5 to 7 mm the return loss gradually increased and less effect on bandwidth. Finally, for 8 mm, the minimum return loss of −50 dB is obtained, which means the maximum possible impedance. 

**Figure 8:**
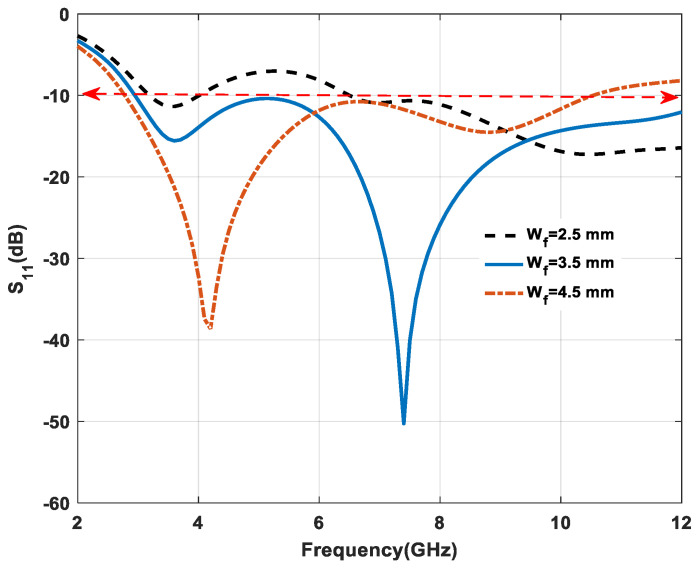
Effect of variation on feed width W_f_.

Figure 8 shows the effect of varying the feed width (W_f_) of microstrip-line-fed on the return loss characteristics. If the values of (W_f_) are increased from 2.5 mm to 4.5 mm, the resonance frequency is shifted towards the lower band. Furthermore, at the 3.5 mm optimum value of (W_f_), the return loss debases at some resonance frequency 7.3 GHz and improves impedance bandwidth. Consequently, an optimum value of 3.5 mm was considered in the proposed prototype. 

## 4. Results and Discussion

**Figure 9:**
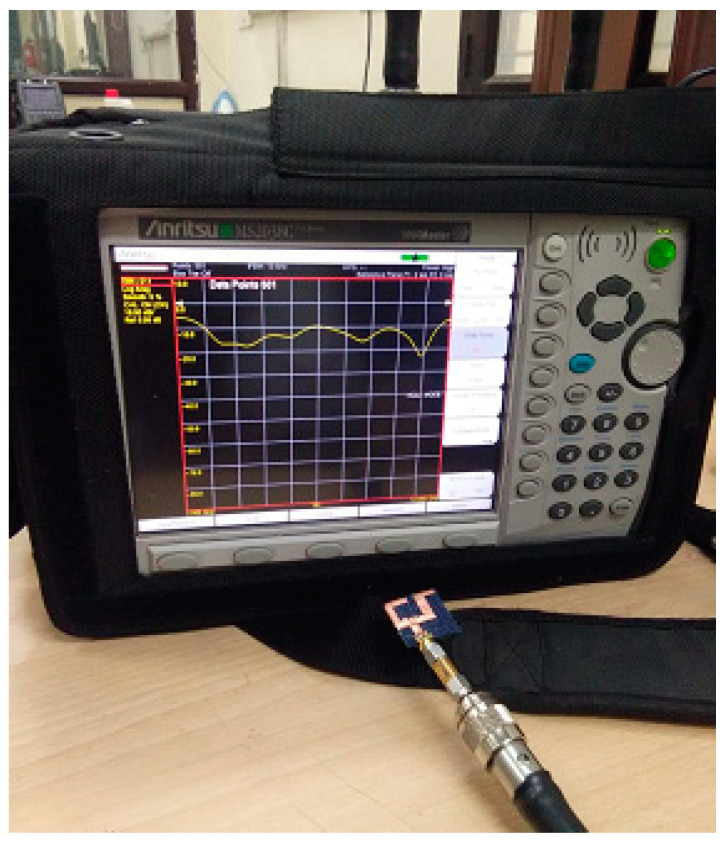
Measurement through the Vector Network Analyzer (VNA Anritsu MS2038C).

**Figure 10:**
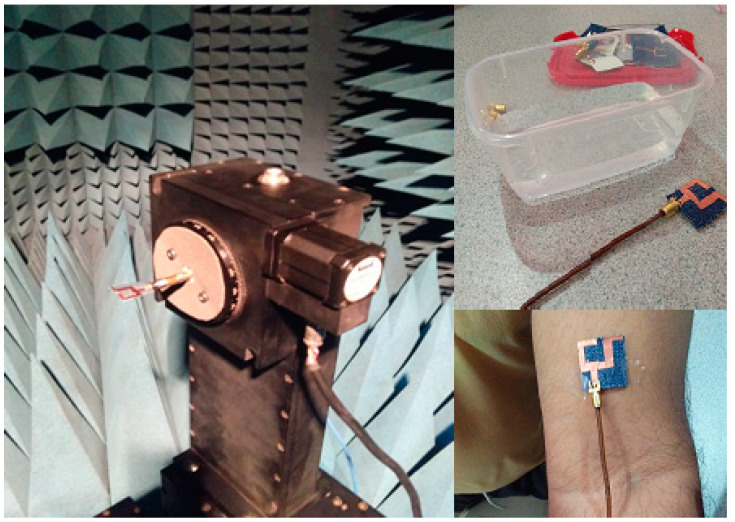
Measurement setup of the anechoic chamber, wet and on body situations.

The presented textile UWB antenna is simulated on HFSS software by using the finite element method (FEM) of simulation. Figure 9 and Figure 10 demonstrate the measurement of the proposed textile antenna through the Vector Network Analyzer (VNA Anritsu MS2038C). 

**Figure 11:**
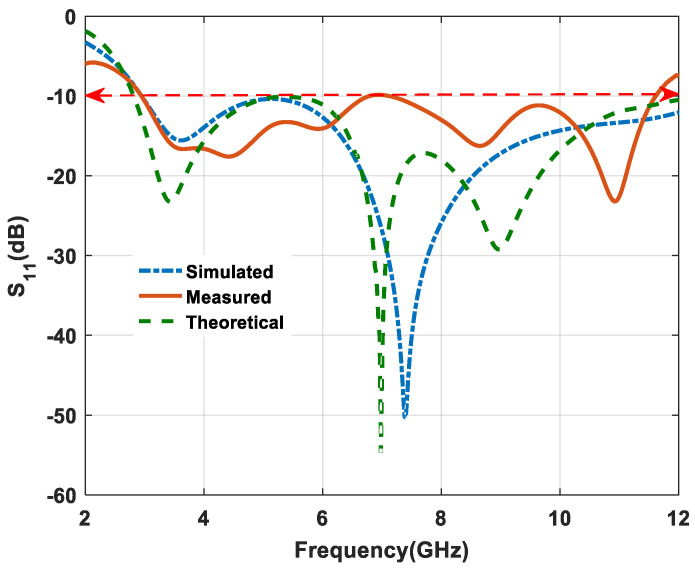
Comparison between S_11_ versus frequency plot.

Furthermore, the comparison of the simulated, measured and theoretical results of return loss (S_11_) is represented in Figure 11. In this figure, it is also evident that the antenna provides an IBW (impedance bandwidth) of 120.85% (2.96–12 GHz) in simulation and approximately 118.68% (2.96–11.6 GHz) in measurement. A significant difference of 2.17% between simulated and measured results was verified. Moreover, theoretical IBW (impedance bandwidth) is 123.88%, which, when compared with measured values, means there is also a significant difference of 5.2%. In the simulation, we have a 100% (ideal) environment, whereas, in practical cases, there are some limitations regarding the fabrication method, material and insertion loss. Due to these limitations, low variations are observed in return loss (S_11_) results of simulation, measurement and theoretical findings.

**Figure 12:**
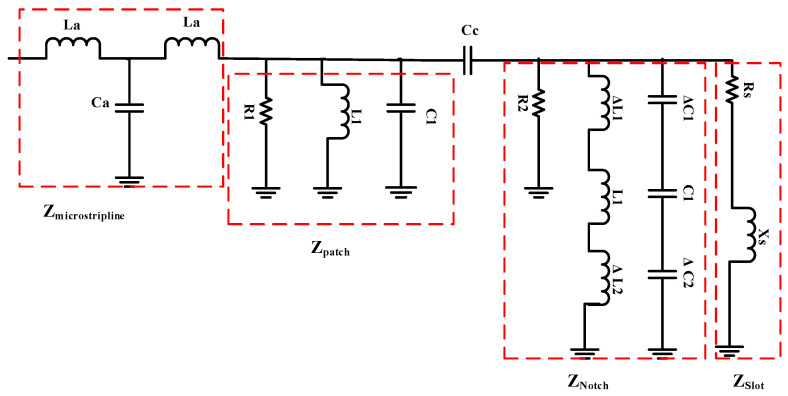
Electrical circuit model of the proposed textile antenna.

The analytical studies were done with the circuit theory concept of the presented electrical equivalent model in Figure 12. The parameters of the electrical circuit model are L_a_ = 0.06 nH, L_b_ = 0.52 nH, C_a_ = 0.81 pF, R_1_ = 183.25 ohm, L_1_ = 1.22 nH, C_1_ = 1.25 pF, R_2_ = 105 ohm, ΔL_1_ = 1.26 nH, ΔL_2_ = 1.33 nH, ΔC_1_ = 0.156 pF, ΔC_2_ = 1.37 pF, R_s_ = 58.5 ohm and X_s_ = 1.07 nH, respectively. 

Furthermore, justifications between both results were also observed. Because of the fabrication challenges of the textile antenna through artwork development and joining the SMA connector, minor variations between both the results (measured and simulated) were obtained.

### 4.1. Gain

**Figure 13:**
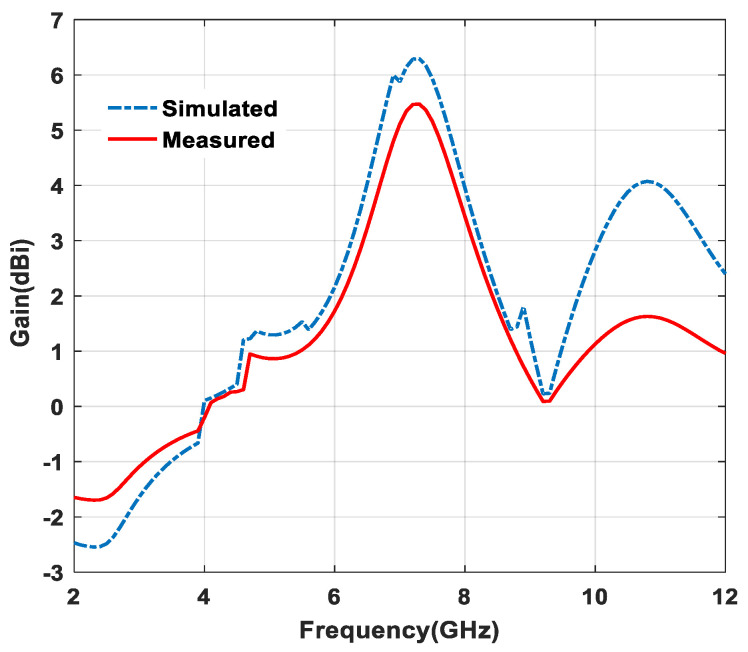
Gain versus frequency plot.

The gain of the antenna is 0 dBi, which means it offers an omnidirectional radiation pattern. On the one hand, when the gain is increasing in positive values, the shape of pattern is changed to directional. On the other hand, when the gain is less than 0 dBi or represents negative values, this means there is a change of shape in radiation pattern due to back lobe variation. Figure 13 depicts the gain of the antenna versus frequency plot, and it also reveals that the highest gain value of 5.47 dBi is observed at 7.3 GHz frequency in broadside direction. It can be noticed that at, specific frequencies, the gain dropped down below 0 dBi, which confirms that the antenna does not radiate energy in the desired direction due to back lobe radiation, whereas gain is almost matched in the other part of the frequencies.

### 4.2. Radiation Pattern

**Figure 14:**
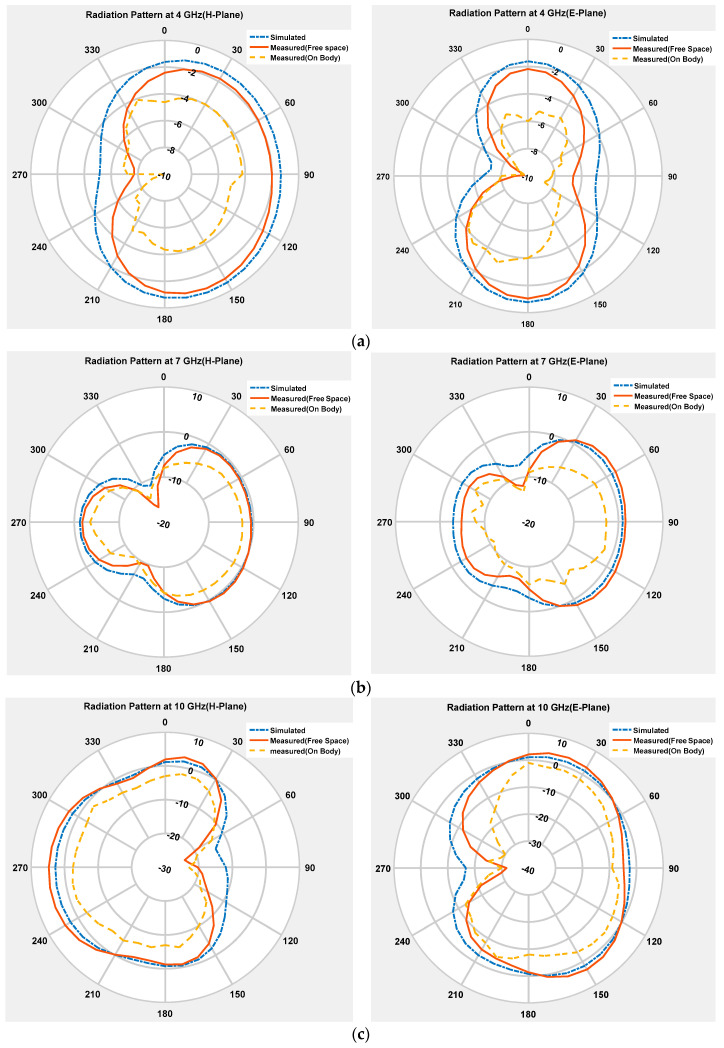
Simulated and measured radiation pattern at 4 GHz (**a**), 7 GHz (**b**) and 10 GHz (**c**) frequencies.

Three radiation pattern plots of the resonating frequencies (4 GHz, 7 GHz and 10 GHz) are illustrated in Figure 14. The H-plane (x-z plane) and E-plane (y-z plane) are selected to explain the antenna radiation patterns. Figure 14 shows the antenna E-field and H-field patterns at 4 GHz, which signifies that an omnidirectional pattern in the H-field and slight variation in E-field pattern has been observed, respectively. Moving towards upper frequencies, at 7 GHz, a quasi-omnidirectional pattern, and, at 10 GHz, omnidirectional patterns in H-planes and E-planes are found, which signifies that the proposed antenna can receive signals from all directions. On the other hand, on-body performance of the antenna is also affected by the lossy component of biological human body tissues, which absorbs the energy from electromagnetic radiation.

### 4.3. Current Distribution

**Figure 15:**
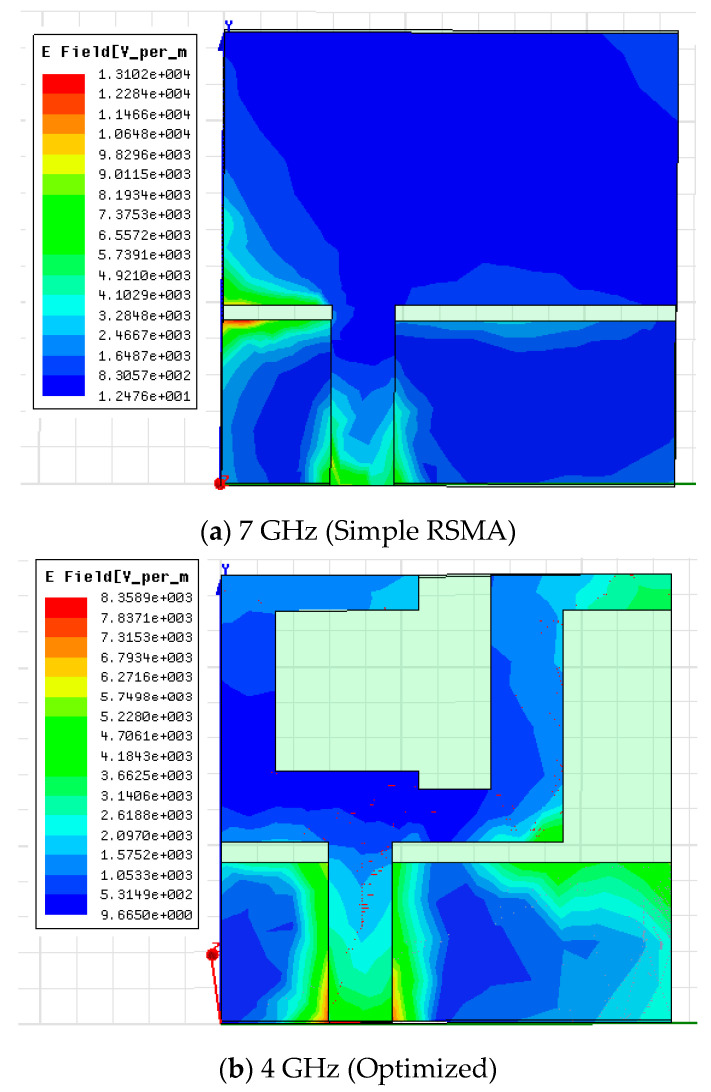

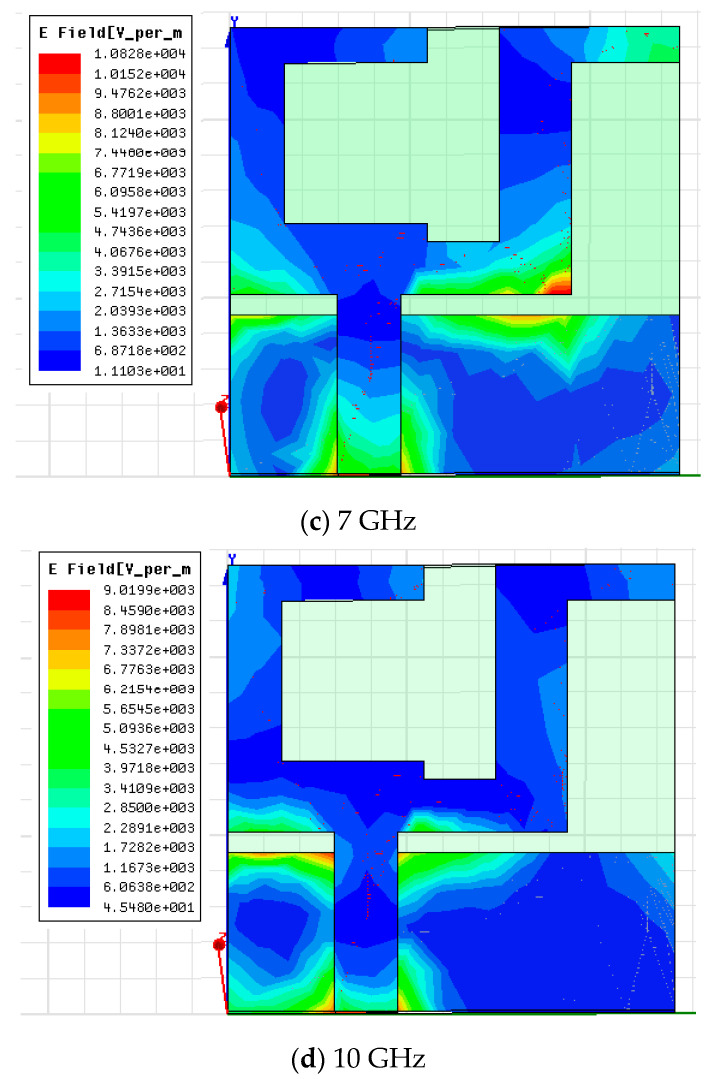
Current distributions at 7 GHz (**a**), 4 GHz (**b**), 7 GHz (**c**) and 10 GHz (**d**) frequencies.

The current distribution effect of the antenna without notch and with notch configuration is explained in Figure 15. Figure 15a shows the effect of current in the primary antenna structure, and it is observed that most of the current has focused around the microstrip line feeding point and left corner of the ground plane. 

Moreover, the distribution of current on the radiator (without a notch) and right corner of the ground are very poor at frequency 7 GHz, which has a significant effect on antenna impedance matching. It can be seen from Figure 15b–d, the current distribution performance has improved by cutting a notch on the edges of a patch from the lower edge of frequency up to the higher edge of frequency of 4 GHz, 7 GHz and 10 GHz, which results in proper impedance matching.

### 4.4. Time Domain Characteristics

**Figure 16:**
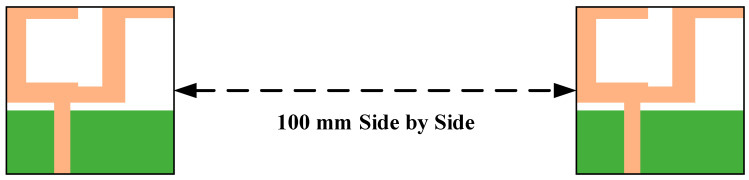
Side-by-side configuration.

**Figure 17:**
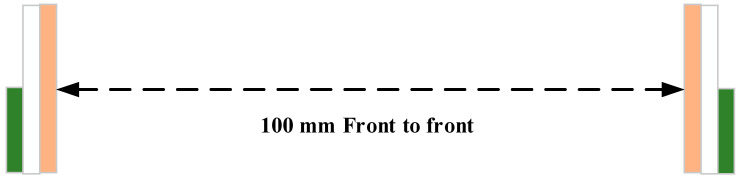
Front-to-front configuration.

The phase response of S_21_, isolation factor and group delay performance were also investigated in transient analysis. The transient analysis was carried out in HFSS simulator by placing two similar antennas, one as a transmitter and other works as a receiver, at a distance of 100 m in side-by-side and front-to-front situations, as shown in Figure 16 and Figure 17. 

**Figure 18:**
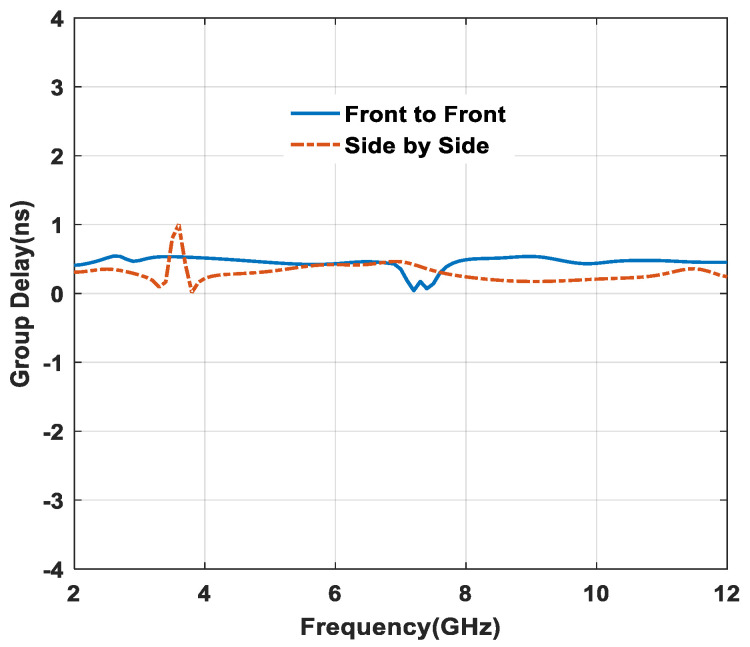
Effect of group delay in distinct configuration.

From Figure 18, it is clear that, approximately constant group delay (i.e., group delay <1 ns) is analyzed for the textile antenna in both side-by-side and front-to-front configurations. Small variations are observed around 3.2 GHz and 7.1 GHz due to small distortions in signal transmission.

**Figure 19:**
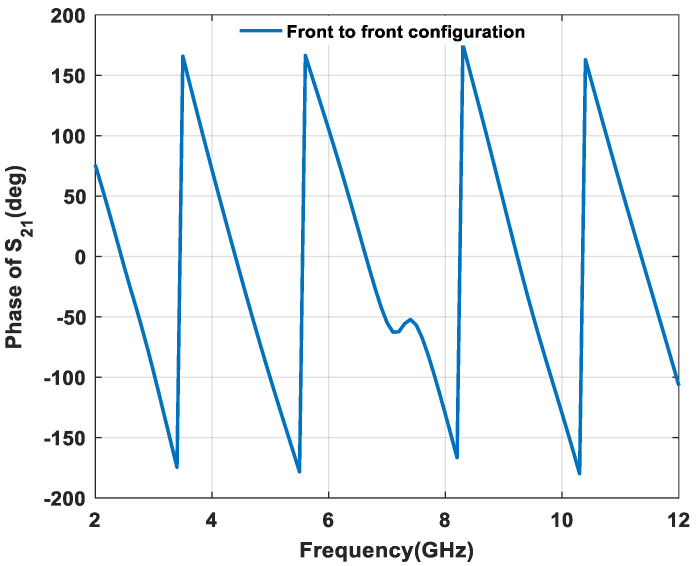
Phase variation effect of s_21_ in front-to-front.

**Figure 20:**
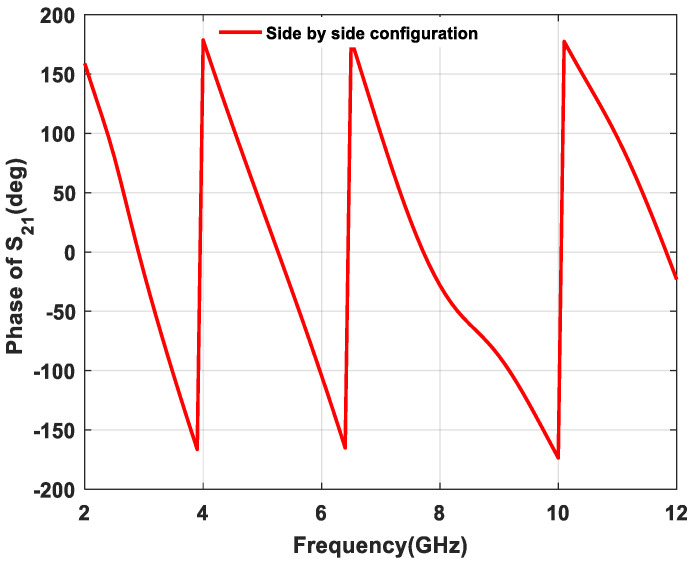
Phase variation effect of s_21_ in side-by-side configuration.

By continuing the second property, the phase response of transmission coefficient S_21_ of the proposed textile UWB antenna is shown in Figure 19 and Figure 20. A linear phase is required to present a good phase characteristic, which reduces the distortion between transmitting and receiving antenna. From Figure 19 and Figure 20, the proposed structure reveals that approximately linear phase response occurred in both front-to-front and side-by-side configurations.

**Figure 21:**
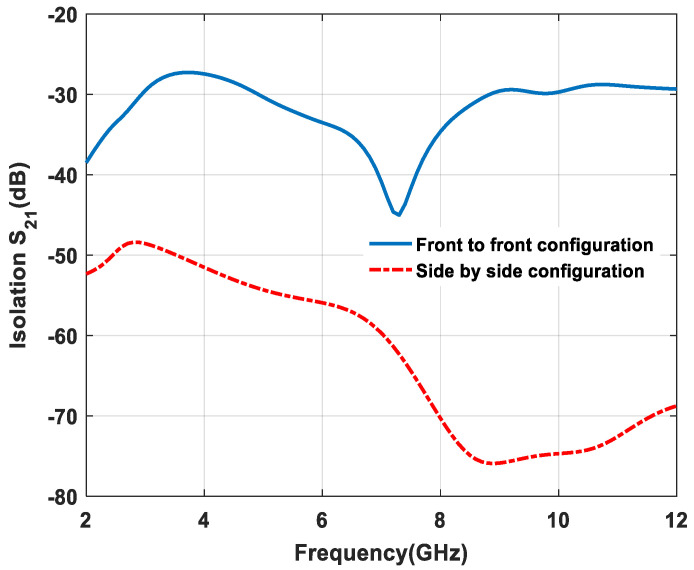
Isolation of S_21_ versus frequency plot.

The isolation characteristic, i.e., S_21_, of the proposed design is also studied and explained in Figure 21. As it can be seen from the plot, the antenna gives proper isolation, i.e., S_21_ < −25 dB, for front-to-front and side-by-side configurations.

### 4.5. Specific Absorption Rate (SAR) Effect on Human Body

For radiation effect assessment on the human body, the antenna is attached with a customized model of the human body in the HFSS environment and the simulations have been performed. The following mathematical formula was used to evaluate SAR value.

(25) $$\mathrm{SAR}=\sigma \frac{{\left|E\right|}^{2}}{\rho }$$

where σ is the conductivity (s/m), E is the electric field (V/m), and ρ is the mass density of biological tissue in Kg/m^3^. The biological properties of human body tissues are given in Table 2 [32,33,34].

**Figure 22:**
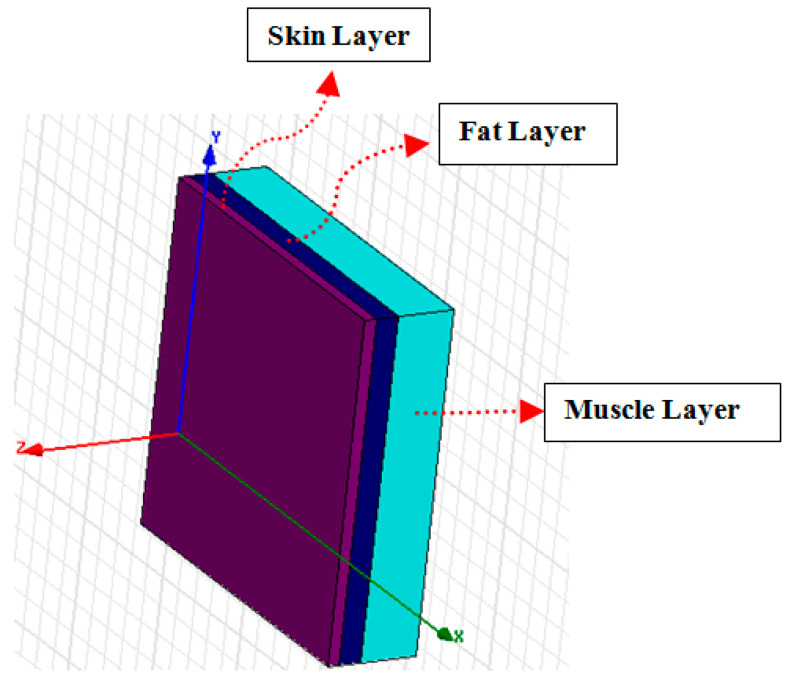
Three-layer human body biological model.

A cross-sectional view of the three-layer human body model is presented in Figure 22. This includes a skin layer, fat layer and muscle layer.

**Figure 23:**
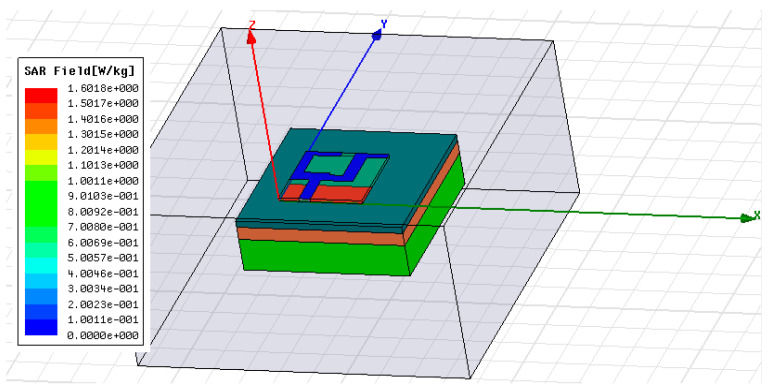
Simulated specific absorption rate (SAR) pattern at 7GHz.

Figure 23 illustrates the SAR simulation at 7 GHz frequency under HFSS environment. From Figure 23 it can be seen that the peak value of a 10 gm averaged and SAR is 1.6018 W/Kg.

By increasing the source power from 25 mW to 150 mW, the SAR value has increased. As per European standard guidelines, the maximum SAR value should not exceed 2 W/Kg of an average mass of 10 gm tissue. The simulated SAR values for different source power inputs are mentioned in Table 3.

### 4.6. Performance Analysis on Human Body

**Figure 24:**
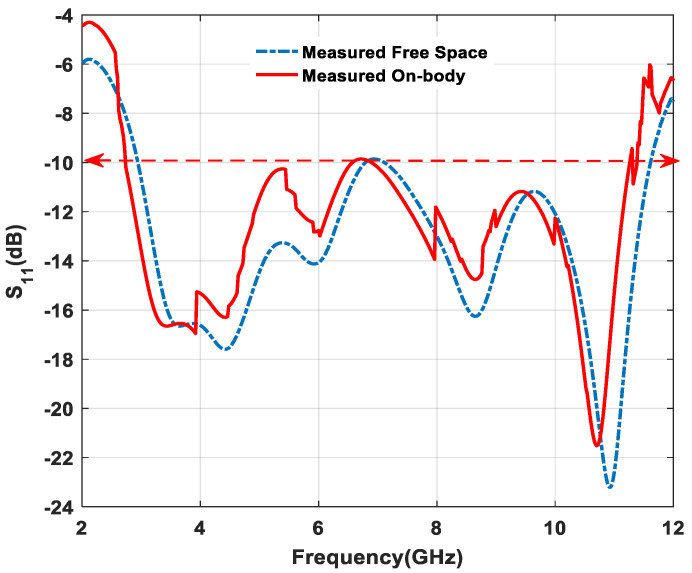
Measured return loss performance for free space and on body.

**Figure 25:**
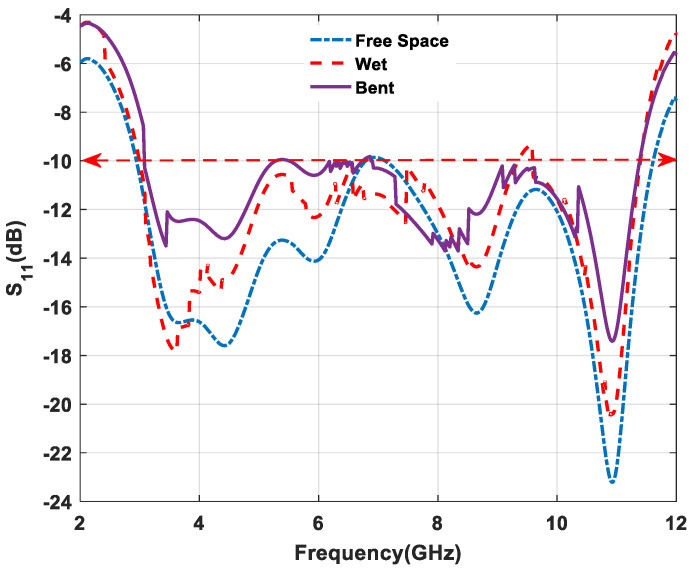
Measured return loss performance under various conditions.

When the antenna is placed on a human body, it is required to work in all situations. However, most of the conventional or rigid antennas have limitations regarding the change of body deformity and dielectric properties of human biological tissues. Textile antennas have a low-profile antenna, i.e., low dielectric constant (ε_r_ = 1.7), and provide reliable impedance matching by reducing surface wave losses. Therefore, textile-based antennas depend on all environmental and other conditions, whereas conventional or rigid antennas do not achieve these requirements [35,36,37]. Figure 24 shows that return loss (S_11_) performance is obtained under the UWB range in free space and on the lower part of the left arm of the human body during measurements. The human body acts as a lossy element due to its biological tissue properties, and it absorbs some amount of the energy from the radiation. Therefore, the return loss obtained during on body measurement is slightly different from the measurement during free space, which is shown in Figure 24. From Figure 25, the antenna is investigated under three different conditions during measurement. The return loss characteristic is varying due to change of subject movement and antenna under wet environmental conditions. After immersing the antenna in water, it was taken out and tested on the VNA. This results in detuning or frequency shift in return loss measurements. The bending of the proposed antenna gives the variation in return loss (S_11_) performance. To show this effect, the antenna was placed on the lower part of the left arm with an angular bending of 10^0^. Measured results are compared with the free-space value and a significant variation of return loss (S_11_) results due to the bending effect is observed.

The comparisons of different UWB antennas are listed in Table 4. Considering the results presented by the authors of [38,39,40,41], the proposed method provides better electrical dimension. Referring to the gain properties and safer SAR limit, the proposed antenna offers better performance than [38,39,40,41].

In summary, the outcomes of this antenna, in comparison with other antennas, are described as follows:

1. The proposed study presents a fabric-substrate-based antenna, which can be integrated with the human body.
2. This textile antenna has low power required to excite the antenna, and low radiation effect.
3. The proposed textile antenna is applicable for high-speed data transfer for short distance coverage.
4. This UWB textile antenna incorporates a compact novel design, which gives novelty of the structure.
5. The electrical equivalent circuit model of the given textile antenna is also presented to calculate the electrical parameters.
6. Excellent time-domain characteristics are achieved to validate the antenna for UWB application. The SAR has also been calculated to avoid the effect of electromagnetic radiation on the human body. In sum, the authors can use various techniques to promote a low SAR limit.

## 5. Conclusions

In this manuscript, the development of compact textile antenna has been designed, which is applicable for the UWB application. The mathematical analysis was completed through circuit theory using the cavity model of antenna, which validates the proposed design. Different slots and notches were removed from the radiating patch, presenting a justified ground plane, and impedance-matching performance through current distribution was presented. 

The proposed antenna has a significant impedance bandwidth of 118.68% (2.96–11.6 GHz), which covers the complete UWB range (3.1–10.6 GHz) designated by the FCC, and has a maximum gain of 5.47 dBi at 7.3 GHz frequency. The time-domain characteristic was also presented to state the pulse distortion phenomena of the developed antenna. The phase of the designed antenna was found to be linear and constant group delay less than 1 ns was achieved. The SAR value of the antenna was tested to show the radiation effect on the human body, and it was found that the SAR value of the antenna is 1.6018 W/Kg, which is less than 2 W/Kg according to the International Electrotechnical Commission (IEC) standard. This point explains the outcomes of this antenna, which distinguishes itself from other antennas. With these results, we propose this wearable textile antenna for telemedicine and mobile health systems.

## Figures and Tables

**Table 1:** Antenna parameters.

S.No.	Parameter	Value (mm)	S.No.	Parameter	Value (mm)
1	W_sub_	25	9	W_2_	8
2	L_sub_	25	10	W_3_	6
3	L_1_	15	11	W_4_	6
4	L_2_	9	12	W_5_	15.5
5	L_3_	13	13	W_6_	4
6	L_4_	2	14	W_f_	3.5
7	L_f_	10	15	G	9
8	W_1_	11	16	h	1

**Table 2:** Specification of human body tissues.

Tissue	Skin	Fat	Muscle
**Permittivity (ε_r_)**	42	5.2853	52.791
**Conductivity (S/m)**	1.5618	0.10235	1.705
**Loss Tangent**	0.2725	0.1450	0.24191
**Density (Kg/m^3^)**	1109	911	1090
**Thickness (mm)**	2	4	10

**Table 3:** Simulated specific absorption rate (SAR) values for different source power inputs.

Frequency (GHz)	Source Power (mW)				
	25	50	75	100	150
**7**	0.7116	1.232	1.4348	1.6018	1.9799

**Table 4:** Comparison of various UWB antennas.

Ref	Substrate	Electrical Dimension	Gain(dBi)	Lower Band Frequency (GHz)	SAR W/Kg
**38**	FR-4, ε_r_ = 4.4	0.23λ_o_ × 0.32λ_o_ × 0.003λ_o_	4.4	2.2	Not reported
**39**	FR-4, ε_r_ = 4.4	0.33λ_o_ × 0.25λ_o_ × 0.013λ_o_	5.9	2.5	Not reported
**40**	ε_r_ = 2.43	0.27λ_o_ × 0.32λ_o_ × 0.004λ_o_	5.4	2.7	Not reported
**41**	FR-4, ε_r_ = 4.4	0.23λ_o_ × 0.29λ_o_ × 0.016λ_o_	2.45	3.1	Not reported
**Proposed**	Jeans, ε_r_ = 1.7	0.24λ_o_ × 0.24λ_o_ × 0.009λ_o_	5.47	2.96	1.6018
